# Tissue Doppler in critical illness: a retrospective cohort study

**DOI:** 10.1186/cc6114

**Published:** 2007-09-06

**Authors:** David J Sturgess, Thomas H Marwick, Christopher J Joyce, Mark Jones, Bala Venkatesh

**Affiliations:** 1 Department of Intensive Care, The Wesley Hospital, Coronation Drive, Brisbane, Queensland, Australia 4066; 2 School of Medicine, University of Queensland, Princess Alexandra Hospital, Ipswich Road, Brisbane, Queensland, Australia 4102; 3 Department of Echocardiography, Princess Alexandra Hospital, Ipswich Road, Brisbane, Queensland, Australia 4102; 4 Department of Intensive Care, Princess Alexandra Hospital, Ipswich Road, Brisbane, Queensland, Australia 4102; 5 School of Population Health, University of Queensland, Princess Alexandra Hospital, Ipswich Road, Brisbane, Queensland, Australia 4102

## Abstract

**Background:**

There is a paucity of published data on tissue Doppler imaging (TDI) in the critically ill. In a critically ill cohort, we studied the distribution of TDI and its correlation with other echocardiographic indices of preload. To aid hypothesis generation and sample size calculation, associations between echocardiographic variables, including the ratio of peak early diastolic transmitral velocity (E) to peak early diastolic mitral annular velocity (E'), and mortality were also explored.

**Methods:**

This retrospective study was performed in a combined medical/surgical, tertiary referral intensive care unit. Over a 2-year period, 94 consecutive patients who underwent transthoracic echocardiography with E/E' measurement were studied.

**Results:**

Mean Acute Physiology and Chronic Health Evaluation III score was 72 ± 25. Echocardiography was performed 5 ± 6 days after intensive care unit admission. TDI variables exhibited a wide range (E' 4.7–18.2 cm/s and E/E' 3.3 to 27.2). E' below 9.6 cm/s was observed in 63 patients (rate of myocardial relaxation below lower 95% confidence limit of normal individuals). Fourteen patients had E/E' above 15 (evidence of raised left ventricular filling pressure). E/E' correlated with left atrial area (*r *= 0.27, *P *= 0.01) but not inferior vena cava diameter (*r *= 0.16, *P *= 0.21) or left ventricular end-diastolic volume (*r *= 0.16, *P *= 0.14). In this cohort, increased left ventricular end-systolic volume, but not E/E', appeared to be an independent predictor (odds ratio 2.1, *P *= 0.007) of 28-day mortality (31%; *n *= 29).

**Conclusion:**

There was a wide range of TDI values. TDI evidence of diastolic dysfunction was common. E/E' did not correlate strongly with other echocardiographic indices of preload. Further evaluation of echocardiographic variables, particularly left ventricular end-systolic volume, for risk stratification in the critically ill appears warranted.

## Introduction

Myocardial dysfunction is common in critically ill patients. Causes include ischaemia, trauma, surgery, sepsis, drugs and toxins. Transthoracic echocardiography is gaining acceptance as a powerful diagnostic tool in this setting [1]. In recent years, tissue Doppler imaging (TDI) has gained increasing acceptance as a means of noninvasively assessing myocardial properties [2] and estimating ventricular filling pressure [3,4], and as a prognostic tool in cardiac diseases [5,6]. However, there is a paucity of published data on TDI in critical illness.

TDI is an echocardiographic technique that measures myocardial velocities [7], which are low frequency, high-amplitude signals filtered from conventional Doppler imaging [8]. The peak early diastolic mitral annular velocity (E'), as measured using TDI, is a relatively preload insensitive assessment of left ventricular relaxation [9]. Although this variable is not independent of large, acute changes in preload (for example, during dialysis [10] or vena caval occlusion [11]), it appears to be less influenced by preload in the critically ill [10]. Also, it does not pseudo-normalize in the same way that transmitral flow does [12]. The influence of changes in ventricular loading on E' in critically ill patients remains incompletely defined [13].

Peak early diastolic transmitral velocity (E) is dependent on left ventricular filling pressure, as well as the rate and extent of left ventricular relaxation [14]. The ratio of E to E' (E/E') has been proposed as an estimate of left ventricular filling pressure that corrects E velocity for the influence of myocardial relaxation [3,4]. There are scant published data regarding the use of TDI in critical care.

The primary aims of this preliminary study were twofold. First, we wished to assess the distribution of values of TDI in critically ill patients. TDI evidence of diastolic dysfunction was accepted as E' below 9.6 cm/s (myocardial relaxation below the lower 95% confidence limit of normal individuals) [15] or E/E' above 15 (mean left ventricular end-diastolic pressure >15 mmHg) [4]. Second, we wished to examine the relationship between TDI (E/E') and other echocardiographic variables. This included left ventricular volumes and alternative indices of ventricular filling pressure such as left atrial size [16] and inferior vena cava (IVC) maximal diameter (right heart) [17].

TDI and other echocardiographic indices have shown prognostic significance in patients with cardiac diseases [5,6,18,19]. No comparable data have been described in the critically ill. This study incorporated a secondary aim of exploring associations between echocardiographic variables, particularly E/E', and mortality. This was undertaken with the intention of hypothesis generation and sample size calculation, with a view to conducting a prospective evaluation in the future.

## Materials and methods

### Patients

Between January 2003 and December 2004 inclusive, 2,695 patients were admitted to the intensive care unit (ICU) of the Princess Alexandra Hospital, Brisbane, Australia, which is an adult medical/surgical tertiary referral ICU. Echocardiography and ICU databases were cross-referenced and yielded a total of 277 clinically requested echocardiograms, performed in 202 patients. Of these, 94 patients included measurement of E/E'. These patients were enrolled. In each case, the first echocardiogram supplemented by measurement of E/E' was studied. Approval for retrospective analysis of clinical data was granted by the Princess Alexandra Hospital Human Research Ethics Committee (protocol number 2005/028).

### Clinical and outcome data

The Acute Physiology and Chronic Health Evaluation (APACHE) III database (Cerner APACHE III^®^; Cerner Corporation, MO, USA) was used to source clinical data, including sex, date of birth, admission and discharge dates, principal reason for ICU admission, ICU and hospital mortality. The APACHE III score and derived risk predictions [20] were also obtained for each patient.

### Echocardiography

All examinations were performed by experienced sonographers using commercially available equipment. Digitally stored images were analyzed by a single observer who was blinded to clinical and outcome data. Measurements were made using AccessPoint™ 2000 software (Freeland Systems, Westfield, IN, USA). Unless otherwise stated, measurements were recorded at end-expiration.

Left ventricular end-diastolic volume (LVEDV) and left ventricular end-systolic volume (LVESV) were calculated using the biplane method of disks (modified Simpson's rule) from the apical four-chamber and two-chamber views [21]. Left ventricular ejection fraction and stroke volume were calculated from LVEDV and LVESV using standard formulae. IVC maximal diameter, independent of respiratory phase, was measured from subcostal views. Zoomed images of the apical four-chamber view were used to measure left atrial (LA) area, and perpendicular LA major (L) and minor (D_1_) axes. LA minor axis (D_2_) was measured from the parasternal long axis view. LA volume was calculated using an ellipsoid model (American Society of Echocardiography guidelines) [21]:

Volume = 4π/3 × (L/2) × (D_1_/2) × (D_2_/2)

Transmitral flow velocities were recorded with pulsed wave Doppler with the sample volume placed at the mitral valve tips from the apical four-chamber view. Peak passive and active velocities were recorded.

Myocardial velocities were obtained using tissue Doppler settings, with the pulsed wave Doppler sample volume at the septal mitral annulus in the apical four-chamber view. Myocardial diastolic velocity (E') was measured and E/E' was calculated.

In the presence of atrial dysrhythmia, transmitral and tissue Doppler velocities were measured over at least five consecutive cardiac cycles.

### Statistical analysis

Analysis was performed by SPSS, version 14.0 for Windows (SPSS Inc., Chicago, IL, USA) and SAS version 9.1 for Windows (SAS Institute, Cary, NC, USA).

Descriptive measures were used to determine the distribution of echocardiographic variables. Differences between groups were assessed using χ^2 ^tests for categorical data. Continuous data were assessed using Levene's test for equality of variance before performing Student's *t*-test for independent samples. Pearson's correlation coefficient was used to examine the relationship between TDI and other echocardiographic variables.

Cox proportional hazards regression was used for time to event outcomes (28-day mortality) from the date of echocardiography. A cut-off *P *value of < 0.1 was used to determine whether predictor variables in univariate models would be selected for inclusion in multiple regression models. A backward elimination procedure was then used to discard all predictor variables with *P *< 0.1 in multiple regression models, one by one, until a final 'best' model was achieved. *P *values relating to survival plots were taken from Log rank tests. In final analyses, *P *< 0.05 was regarded as significant. Unless stated otherwise, results are reported as mean ± standard deviation.

## Results

### Patient characteristics

The study cohort consisted of 28 females (30%) and 66 males (70%), with a mean age of 61 ± 15 years. Transthoracic echocardiography was performed a mean of 5 ± 6 days from ICU admission (61% within 3 days of ICU admission). Inspection of data (Table 1) reveals that the study cohort had a higher severity of illness than that in the general ICU population during the same period. On the day of echocardiography, 37 out of the 94 patients were mechanically ventilated. At the time of echocardiography, atrial fibrillation was present in four (4%) participants. None had atrial flutter.

**Table 1:** Demographic data of the study cohort and all ICU patients between January 2003 and December 2004

Characteristic	Cohort (*n *= 94)	ICU patients (*n *= 2,695)	*P*
Female sex	28 (31%)	808 (30%)	≤1
Age (years)	61 ± 15	58 ± 17	0.065
APACHE III score	72 ± 25	53 ± 25	≤0.001
Length of ICU stay (days)	11.5 ± 11	3.5 ± 6.5	≤0.001^a^
Length of hospital stay (days)	32 ± 48	19 ± 29	0.01^a^
ICU mortality	22 (23%)	201 (7.5%)	≤0.001
Hospital mortality	31 (33%)	309 (11.5%)	≤0.001

Results are expressed as mean ± standard deviation or number (percentage). ^a^Unequal variance assumed (Levene's test *P *< 0.001). APACHE, Acute Physiology and Chronic Health Evaluation; ICU, intensive care unit; database.

### Echocardiography

Echocardiographic characteristics of the cohort are presented in Table 2. Values of E' ranged from 4.7 to 18.2 cm/s, with 67% (*n *= 63) demonstrating impaired myocardial relaxation (E' < 9.6 cm/s). In the absence of defined reference ranges for the critically ill, a cut-off of 9.6 cm/s was accepted. This represents the lower 95% confidence limit for segmental E' in normal individuals [15]. Based on the E/E' ratio alone, 26 patients demonstrated normal left ventricular filling pressure (E/E' < 8) whereas 14 had raised filling pressure (E/E' > 15) [4]. The remaining 54 patients had E/E' in the intermediate range.

**Table 2:** Echocardiographic characteristics of patients

Characteristic	Cohort (*n *= 94)	Reference range
E (m/s)	0.89 (0.3 to 1.9)	0.44 to 1.0
A (m/s)	0.79 (0.3 to 2)	0.2 to 0.6
E' (cm/s)	8.8 (4.7 to 18.2)	9.6 to 11
E/E' ratio	10.96 (3.3 to 27.2)	<8
LA area (cm^2^)	24 (8 to 40)	≤20
LA volume (ml)	57 (10 to 99)	22 to 58
LVEDV (ml)	129 (42 to 378)	56 to 155
LVESV (ml)	74 (5 to 264)	19 to 58
LV stroke volume (ml)	54 (10 to 137)	
LV ejection fraction (%)	47 (7 to 93)	≥55
IVC diameter (cm)	2.1 (1.1 to 3.3)	<1.7

Results are expressed as mean (range). Reference ranges are not specific for critically ill patients [4,15,21,33]. A, peak active transmitral velocity; E, peak early diastolic (passive) transmitral velocity; E', peak early diastolic mitral annular velocity; IVC, inferior vena cava; LA, left atrial; LV, left ventricular; LVEDV, left ventricular end-diastolic volume; LVESV, left ventricular end-systolic volume.

There was no significant difference in the value of E' between ventilated and nonventilated patients (8.8 ± 2.9 cm/s versus 8.8 ± 3 cm/s, respectively; *P *= 0.9 [equal variance assumed; Levene's test *P *= 1.0]). Likewise, the value of E/E' did not differ significantly between ventilated and nonventilated patients (11.1 ± 4.5 versus 10.7 ± 4.6, respectively; *P *= 0.7 [equal variance assumed; Levene's test *P *= 0.89]). The mechanically ventilated group exhibited an increased IVC maximal diameter compared with the nonventilated group (2.3 ± 0.5 cm versus 1.9 ± 0.5 cm, respectively; *P *= 0.015 [equal variance assumed; Levene's test *P *= 0.88]).

When all patients were included, there were no significant correlations between E' and the other echocardiographic variables (other than E/E' ratio). Subgroup analysis of patients who were mechanically ventilated on the day of echocardiography revealed a correlation between E' and heart rate (*r *= 0.265, *P *= 0.048).

The correlation between E/E' and other echocardiographic indices of preload were as follows: E/E' ratio versus LA area, *r *= 0.27 (*P *= 0.01); E/E' ratio versus LVEDV, *r *= 0.16 (*P *= 0.14); and E/E' ratio versus IVC diameter, *r *= 0.16 (*P *= 0.21). In mechanically ventilated patients, the correlation between E/E' and LA area was significant (*r *= 0.3, *P *= 0.026); however, this relationship was not observed in the nonventilated group (*r *= 0.21, *P *= 0.22).

### Associations with mortality

The all-cause ICU mortality rate was 23%, and corresponding 28-day and hospital mortality rates were 31% and 33%, respectively.

**Figure 1:**
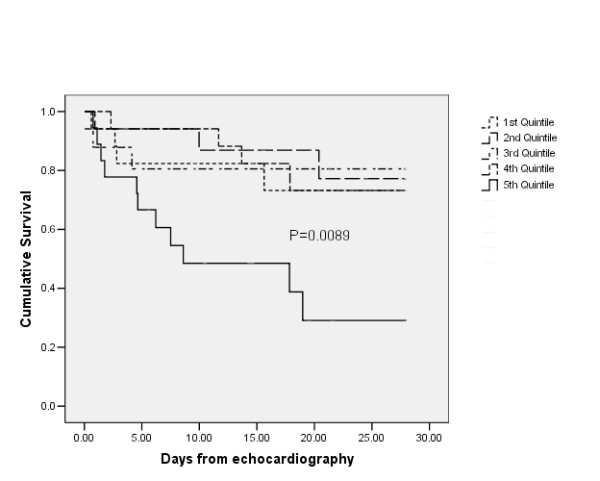
Survival at 28 days. Shown is a Kaplan-Meier curve of 28-day survival, according to quintiles of left ventricular end systolic volume (*P *= 0.0089). Threshold values (ml): first quintile ≤ 27; second quintile > 27 but ≤ 45; third quintile > 45 but ≤ 72; fourth quintile > 72 but ≤ 105; and fifth quintile > 105. Log rank analysis confirms no significant difference between survival curves for the first to fourth quintiles (*P *= 0.97).

Univariate analysis yielded significant associations between 28-day mortality and APACHE III predicted hospital death, LVEDV and LVESV (Table 3). In this cohort, LVESV was also an independent predictor of mortality. The resultant odds ratio suggests that the risk for death approximately doubles for each 100 ml increase in LVESV. However, log rank analysis reveals that only LVESV greater than 105 ml (highest quintile) was associated with a significantly different Kaplan-Meier curve (Figure 1).

**Table 3:** Clinical and echocardiographic correlates of 28-day mortality

Variable	Univariate		Multiple regression	
	
	OR (95% CI)	*P*	OR (95% CI)	*P*
Female sex	0.85 (0.38 to 1.9)	0.7		
Age (per decade)	1.03 (0.8 to 1.3)	0.8		

APACHE III predicted hospital death (×10)^a^	1.18 (1.03 to 1.4)	0.017	1.3 (1.1 to 1.5)	0.0028

E (m/s)	2.3 (0.7 to 7.6)	0.16		
A (m/s)	0.74 (0.22 to 2.5)	0.6		
E' (×10 cm/s)^a^	1.0 (0.5 to 1.9)	0.9		
E/E' ratio (/10)^a^	1.3 (0.6 to 2.8)	0.5		
LA area (/10 cm^2^)^a^	1.5 (0.8 to 2.9)	0.19		
LA volume (/100 mL)^a^	1.4 (0.22 to 9)	0.7		
LVEDV (/100 mL)^a^	2.0 (1.2 to 3.3)	0.0059		
LVESV (/100 mL)^a^	2.2 (1.3 to 3.8)	0.0047	2.1 (1.2 to 3.7)	0.0068
LV stroke volume (/100 mL)^a^	1.4 (0.32 to 6.1)	0.7		
LV ejection fraction (/100%)^a^	0.24 (0.03 to 1.7)	0.15		
IVC diameter (cm)	1.8 (0.8 to 4.1)	0.14		

For multiple regression analysis, only variables included in the final best model are shown. ^a^The scale of these variables was altered by the amount shown in parentheses to aid interpretation of odds ratios. A, peak active transmitral velocity; APACHE, Acute Physiology and Chronic Health Evaluation; CI, confidence interval; E, peak early diastolic (passive) transmitral velocity; E', peak early diastolic mitral annular velocity; IVC, inferior vena cava; LA, left atrial; LV, left ventricular; LVEDV, left ventricular end-diastolic volume; LVESV, left ventricular end-systolic volume; OR, odds ratio.

Comparison between survivors and nonsurvivors at 28 days revealed no significant differences in E' (8.7 ± 2.7 cm/s versus 9.1 ± 3.5 cm/s, *P *= 0.58), E/E' ratio (10.8 ± 4.8 versus 11.4 ± 3.9, *P *= 0.5), LA area (23.3 ± 6.4 cm^2 ^versus 25.6 ± 6.7 cm^2^, *P *= 0.14), or IVC maximal diameter (2.0 ± 0.5 cm versus 2.2 ± 0.5 cm, *P *= 0.13).

## Discussion

This study was performed in response to increasing utilization of echocardiography in our ICU. TDI is routinely performed by our echocardiographers as part of a comprehensive transthoracic echocardiography examination. Although there are increasing data supporting the role of TDI in clinical cardiology [12], there are scant data regarding its application to critical care.

In this study of critically ill patients, the clinical decision to perform echocardiography selected a cohort with high severity of illness (mean APACHE III score 72). A wide range of echocardiographic values were observed. Extreme values, such as LVESV of 5 ml and left ventricular ejection fraction of 7%, reflect the high severity of illness.

The cardinal findings of this study were as follows. First, there was a wide range of E' values (4.7 to 18.2 cm/s), with a mean of 8.8 cm/s. Approximately two-thirds of the cohort exhibited TDI evidence of delayed myocardial relaxation (E' < 9.6 cm/s). Second, there was a wide range of E/E' ratios (3.3 to 27.2). The mean value (10.96) is within the intermediate range for left ventricular end-diastolic pressure. Of the cohort, 15% (*n *= 14) demonstrated Doppler evidence of elevated left ventricular filling pressure (E/E' > 15). Third, there was a weak correlation between E/E' and LA area. On subgroup analysis, this correlation persisted only in the mechanically ventilated patients. No correlations were demonstrated between E/E' and LA volume, IVC diameter, or LVEDV. Finally, in the selected cohort, increased LVESV, but not E' or E/E', was associated with excess 28-day mortality.

In this preliminary retrospective study, we were able to define a range of TDI values for a cohort of critically ill patients. This extends previously published data. TDI evidence of diastolic dysfunction was common in this critically ill cohort. There was TDI evidence of impaired myocardial relaxation in two-thirds of the patients, and elevated left ventricular filling pressure in 15% or more.

Robust diagnosis of diastolic dysfunction is difficult regardless of the method of evaluation [22]. Although cardiac catheterization and measurement of intracardiac pressures allow analysis of pressure-volume loops and rates/time constants of pressure change, these techniques are impractical in critical care. Echocardiography, on the other hand, is a readily available bedside tool. It is safe in critically ill patients and is increasingly accepted in their care [1].

Although TDI is not independent of large, acute changes in preload (for example, during dialysis [10] or vena caval occlusion [11]), it appears to be less influenced by preload in the critically ill [10]. Furthermore, it does not pseudo-normalize in the same way that transmitral flow does [12]. The influence of changes in ventricular loading on E' in critically ill patients remains incompletely defined [13]. Thus, it is not possible to assert its preload independence in this setting. We report TDI and Doppler evidence of diastolic dysfunction, rather than a diagnosis of diastolic dysfunction *per se*.

We are unaware of any previously published correlations between echocardiographic indices of ventricular filling in critically ill patients. Because of anticipated feasibility and ease of measurement in the critically ill, we chose to compare E/E' with LA size (area and volume) and IVC maximal diameter. The lack of good correlation between these variables probably reflects the different elements of ventricular filling that each represents. The E/E' ratio, derived from conventional Doppler and TDI, has been proposed as an estimate of left ventricular filling pressure [3,4]. This has been validated in a wide range of clinical settings, including critical illness [23,24] and atrial fibrillation [25]. LA dimensions are more stable than Doppler velocities, thus reflecting the duration and severity of diastolic dysfunction [26]. IVC diameter was included as a readily measured estimate of right ventricular filling even though it appears to be less robust in mechanically ventilated patients [21].

The lack of correlation between these indices of ventricular filling pressure and LVEDV probably reflects the heterogeneity of myocardial compliance that is commonly observed in critically ill patients [22].

Increased LVESV has been documented to be a predictor of mortality in other clinical settings [27,28]. It may be a marker of severe myocardial dysfunction, and therefore poor prognosis, independent of underlying pathology. LVESV is a complex variable that is determined by the interaction of preload, afterload, and contractility. These factors are frequently manipulated in ICU or are affected by underlying pathology (such as dilated cardiomyopathy). In the current cohort, only the highest quintile (>105 ml) demonstrated significantly different survival. It was not possible to assess the contribution of therapy or underlying pathology.

E' and E/E' were not predictors of mortality in the selected cohort. This differs from other published data [5,6,29-31]. The lack of association of TDI (E' and E/E') with outcome may attest to these signals being influenced by therapeutic measures as much as being markers of underlying disease. This is an important consideration in evaluating TDI as a prognostic indicator in the critically ill. Prospective evaluation should account for haemodynamic status and concurrent therapeutic intervention. Another consideration is the potential prognostic relevance of changes in these variables over time. For instance, worsening diastolic function despite appropriate therapy might be a more sensitive indicator of unfavourable prognosis.

Another consideration for prospective evaluation is sample size calculation. Accepting a 28-day mortality of 31% and α of 0.05, the number of nonsurvivors required to achieve 80% power was calculated for the following variables [32]: E' 1,136 nonsurvivors (difference between means [δ] = 0.3, standard deviation [σ] = 3); E/E' 429 nonsurvivors (δ = 0.7, σ = 4.3); LA area 90 nonsurvivors (δ = 2.32, σ = 6.5), and IVC maximal diameter 65 nonsurvivors (δ = 0.21, σ = 0.5).

### Study limitations

The cohort presented here represents a consecutive group of patients in whom E/E' was performed on clinical grounds, thus increasing the potential for selection bias. Echocardiography was not routinely performed at the time of hospital or ICU admission. It is likely that the results would be influenced by timing of echocardiography relative to initiation and progress of therapy.

This study incorporated a secondary aim of exploring associations between echocardiographic variables, particularly E/E', and mortality. It is unlikely that any isolated echocardiographic measurement taken at a variable point in the disease/treatment process will contribute to risk stratification. However, this important limitation was accepted with the intention being to generate hypotheses that can be tested prospectively. Timing of echocardiography and concurrent interventions should be considered in planning prospective evaluation.

Despite these methodological issues, the novel aspects of the study include the generation of potential reference ranges for TDI indices in critically ill patients, which can provide a framework for planning future studies. The findings of this retrospective, single centre study should be confirmed by a larger, prospective and multicentre study.

## Conclusion

This critically ill cohort exhibited a wide range of TDI values. Diastolic dysfunction, as evidenced by TDI, was common in this critically ill cohort. E/E' did not correlate strongly with other echocardiographic indices of preload. Further evaluation of echocardiographic variables, particularly increased LVESV, for risk stratification in the critically ill appears warranted.

## Key messages

• There was a wide range of E' value (mean 8.8 cm/s; range 4.7 to 18.2 cm/s) and E/E' ratios (mean 10.96, range 3.3 to 27.2).

• Approximately two-thirds of the cohort exhibited TDI evidence of delayed myocardial relaxation (E' < 9.6 cm/s).

• Fifteen percent (*n *= 14) of the cohort demonstrated Doppler evidence of elevated left ventricular filling pressure (E/E' > 15).

• There was a weak correlation between E/E' and LA area, which persisted only in the mechanically ventilated patients; no correlations were demonstrated with LA volume, IVC diameter, or LVEDV.

• In the selected cohort, increased LVESV, but not E' or E/E', was associated with excess 28-day mortality.

## Abbreviations

APACHE = Acute Physiology and Chronic Health Evaluation; E = peak early diastolic transmitral velocity; E' = peak early diastolic mitral annular velocity; ICU = intensive care unit; IVC = inferior vena cava; LA = left atrial; LVEDV = left ventricular end-diastolic volume; LVESV = left ventricular end-systolic volume; TDI = tissue Doppler imaging.

## Competing interests

The authors declare that they have no competing interests.

## Authors' contributions

DS conceived of the study, and participated in its design and coordination, and drafted the manuscript. TM conceived of the study, and participated in its design and helped to draft the manuscript. CJ participated in the design of the study and helped to draft the manuscript. MJ participated in the design of the study and performed the statistical analysis. BV conceived of the study, and participated in its design and helped to draft the manuscript. All authors read and approved the final manuscript.
